# Healthcare professionals’ experiences of providing palliative care for patients with diabetes – a qualitative study

**DOI:** 10.1186/s12904-024-01567-4

**Published:** 2024-10-10

**Authors:** Signe Seim, Ragnhild Elisabeth Monsen, Beate-Christin Hope Kolltveit, Marit Graue

**Affiliations:** 1 Department of Health and Caring Sciences, Faculty of Health and Social Sciences, Western Norway University of Applied Sciences, P.O. Box 7030, Bergen, NO-5020 Norway; 2 Nesodden municipality, Nesodden, Norway; 3 Lovisenberg Diaconal Hospital, Oslo, Norway

**Keywords:** Diabetes, Palliative care, End-of-life care, Quality of life, Qualitative interviews

## Abstract

**Background:**

At present, there are no specific guidelines for the treatment of diabetes in palliative care in Norway. The aim of this study was therefore to explore healthcare professionals’ experiences of providing palliative care to individuals with diabetes in specialist as well as primary care settings.

**Methods:**

We interviewed 12 healthcare professionals from two palliative care units in specialist healthcare, one hospice unit in a nursing home, and one dietary care unit providing counselling in the municipality in the eastern part of Norway. Thematic analysis was used to analyze the data.

**Results:**

Our analysis generated three main themes: 1) “Quality of life is the main focus”, which showed that the healthcare professionals’ main focus was on comforting patients through engagement and communication; 2) “An individualized approach”, emphasizing that the treatment was tailored to the unique circumstances of each individual and considered factors such as life expectancy, difficult blood glucose control, and multidisciplinary collaboration, and 3) “Diabetes in the background”, which highlighted that they had a modest focus on diabetes. Diabetes was seen as another aspect of health that they had to be aware of, but their limited knowledge of diabetes guidelines, technical tools, and treatment choices underscored that attentiveness to the diabetes treatment was not prominent.

**Conclusion:**

The findings show that a lack of guidelines allowed for diverse approaches to the treatment of patients with diabetes in palliative care. Attentiveness to diabetes was based on the individual healthcare professionals’ experience and expertise, professional views, and the circumstances of each individual.

**Supplementary Information:**

The online version contains supplementary material available at 10.1186/s12904-024-01567-4.

## Background

Quality of life is defined by the World Health Organization (WHO) as: “An individual’s perception of their position in life in the context of the culture and value systems in which they live and in relation to their goals, expectations, standards and concerns” [1]. Palliative care is an approach aimed at improving the quality of life of patients who have a life-threatening disease [2]. According to the WHO, an estimated 56.8 million people require palliative care, and 34% of these individuals have been diagnosed with cancer [2]. The largest proportion of patients who receive palliative care in Norway today are cancer patients. Because of modern cancer treatment, patients live longer, and other underlying diseases must also be followed up [3]. Among patients diagnosed with breast, lung, prostate, and colon cancer, more than 80% reported at least one comorbid disease [4].

Diabetes is a worldwide health challenge, and it is estimated that over 780 million people will be affected by 2045 [5]. The disease affects about 8.5% of the adult population worldwide [6]. The prevalence of diabetes among cancer patients ranges from 8 to 18% [4]. In general, patients with diabetes are encouraged to meet specific agreed treatment goals in order to avoid or delay complications and improve their general wellbeing [7]. In patients with cancer, treatment of the cancer itself may overshadow a non-cancer diagnosis such as diabetes [5]. In addition, people with diabetes often have other chronic diseases, such as high blood pressure, cardiovascular disease, kidney disease, and neurological complications, which add to the advanced and complex state of disease. For patients with advanced cancer and diabetes, a shift may occur in the focus, from strict glycemic control to preventing symptoms of hypoglycemia and hyperglycemia [8, 9]. Providing care for patients with diabetes in palliative care imply that the treatment needs to be tailored to the unique circumstances of each individual taking difficult blood glucose control into consideration regardless of type of diabetes.

Diabetes research conducted in palliative care settings is sparse, and mostly comprises research of low methodological quality or expert opinions [10, 11]. The aim of this study was therefore to explore healthcare professionals’ experience of treating patients with diabetes in palliative care from the perspective of nurses, physicians, and dietitians.

## Methods

This was a qualitative study conducted in specialist and primary care settings. The study is reported in accordance with the COREQ (Consolidated Criteria for Reporting Qualitative Research) Checklist [12].

### Study participants and recruitment

Participants were recruited from four palliative care units located in the eastern part of Norway. They comprised specialized palliative care units in two hospital settings, one hospice unit in a nursing home, and one dietary care unit providing counselling in the municipality. These settings are mainly providing palliative care in late stage and end-of-life stage. The inclusion criteria were nurses, physicians, and dietitians who had worked for more than six months in palliative care, had more than six months’ experience of caring for patients with diabetes in a palliative setting, and were able to communicate in both written and oral Norwegian. Participants with less than one year of experience as healthcare providers were excluded. Potential participants were approached by their managers, who informed them about the study and invited them to participate.

### Data collection

Based on the aim of the study, a semi-structured interview guide (Suppl file 1) was developed by the first author (SS), a female diabetes nurse specialist, in collaboration with another diabetic nurse specialist and one cancer coordinator. The last author (MG), who is a Professor of Nursing at the university, and one palliative care nurse specialist (REM) provided input in connection with finalization of the guide. The interview guide was used to enable participants to respond in their own words, thereby emphasizing their views. A pilot interview was conducted with one nurse in specialist care, which resulted in some adjustments to the interview guide. The pilot interview was not included in the analysis.

We conducted interviews with eleven women and one man. Eight participants were nurses, two were physicians and two dietitians. The participants had worked with patients in a palliative care setting for from three to eighteen years. A schematic representation of the workplace, further education, or specialization is shown in Table 1.

**Table 1:** Schematic presentation of the participants’ workplace, further education, and specialization

Profession	Workplace	Further education/specialization
Physician	The primary healthcare service	Specialist in general medicine
Physician	The specialist healthcare service	Specialist in general internal medicine
Dietitian	The primary healthcare service	PhD
Dietitian	The specialist healthcare service	No further education
Nurse	The specialist healthcare service	Further education in palliative care
Nurse	The specialist healthcare service	Further education as specialist cancer nurse
Nurse	The specialist healthcare service	Further education as specialist cancer nurse
Nurse	The specialist healthcare service	Further education as specialist cancer nurse
Nurse	The specialist healthcare service	Further education as specialist intensive care nurse
Nurse	The specialist healthcare service	No further education
Nurse	The primary health care service	Furter education in family therapy
Nurse	The primary healthcare service	No further education

All the interviews were conducted face-to-face by the first author. The participants chose the location of the interviews. Most interviews were conducted at the participants’ workplace during their working hours, except for one interview which took place in the home of the participant. The interviews were performed by the first author between September 2022 and January 2023 and lasted for from 17 to 77 min (average 46.5 min). They were audio recorded using a digital voice recorder and transcribed verbatim by the first author (SS).

### Data analysis

The interviews were analyzed following the six steps of thematic analysis described by Braun and Clarke [13–15] (Table 2).

**Table 2:** The six steps of thematic analysis (Braun & Clarke 2021)

Step 1	Familiarisation with the data
Step 2	Generating initial codes
Step 3	Searching for themes
Step 4	Reviewing themes
Step 5	Defining and naming themes
Step 6	Producing report

Thematic analysis is theoretically flexible. It focusses on identifying, analyzing, and reporting patterns of meaning across a dataset [13]. When conducting a thematic analysis, the researcher continuously moves back and forth between the different steps, uses open coding to develop themes, and revises them in all steps of the analysis [13–15].

Initially, all researchers read all the transcribed data several times to familiarize themselves with the data. Then each author noted down their preliminary thoughts and generated codes. The study team met for three workshops. At the first meeting, the first author shared her initial thoughts and preliminary codes. The other researchers provided feedback and added to the discussion by sharing their codes to reduce researcher bias. At the second workshop, all four researchers shared their thoughts on the data material that were relevant to the aim of the study, assessed codes by pattern, and discussed how different codes could be combined to form themes. At the third workshop, all the authors reviewed and discussed the themes presented by the first author and shared their reflections on creating new codes and themes. The group reflected on data that fell outside the previous coding. The first author then continued the process and drafted the paper.

### Ethical considerations

The study was conducted in accordance with the Declaration of Helsinki. All participants gave informed written consent to participate in the study and to the recording. They were informed that they could withdraw from the study at any time without giving a reason. An application was sent to the Regional Ethics Committee for Human Research (REK), which assessed the project as not requiring notification (ref. 492437). The project was then approved by the Data Protection Officer at AHUS hospital and Lovisenberg Diaconal Hospital, and the Norwegian Agency for Shared Services in Education and Research (SIKT) (ref. 550782).

## Results

The analysis revealed three main themes in the healthcare professionals’ experiences of providing palliative care for patients with diabetes. The first, “Quality of life is the main focus”, shows that the healthcare professionals’ main focus was on comforting patients through engagement and communication. The second, “An individualized approach”, emphasizes that the treatment was tailored to the unique circumstances of each individual and took factors such as life expectancy, difficult blood glucose control, and multidisciplinary collaboration, into consideration. Finally, “Diabetes in the background” showed that the healthcare professionals only expressed a modest focus on diabetes. The need for vigilance in managing diabetes was recognized as an additional dimension of healthcare, but limited knowledge of diabetes guidelines, technical tools, and treatment options highlighted the insufficient emphasis on diabetes treatment.

### Quality of life is the main focus

The main focus of the healthcare professionals was on comforting patients by optimizing their health and satisfaction through engagement and communication aimed at improving their quality of life. They tried to adapt to the patient’s dietary wishes and emphasized that this way of working is necessary to provide the patients with holistic care. Having enough time for conversations between patients and the healthcare professionals was a key issue in comforting the patients. One nurse expressed this as follows:

*Quality of life is about so many things*,* you know. If you serve an appealing plate of good food*,* that’s definitely quality of life. It means that they have the necessary energy and have an appetite*,* for example; the opposite is people who feel nauseous and throw up … that’s maybe the worst case*,* but*,* yes*,* things like that. Or you sit down and talk to them*,* show some interest in their children and grandchildren and are interested in who they are and what they did before and*,* yes*,* lots of things like that. Serve a glass of wine to someone who wants one*,* there’s a lot of quality of life in a little alcohol* (Participant 10).

An important part of supporting the patient’s quality of life was to allow the patients to show emotions and cry. In addition, calmness, having enough time and opportunity to listen to the patients, and allowing the patients to take breaks in the conversations was experienced by the healthcare professionals as important to promote quality of life. They also noted that engaging with the patient’s family would benefit the patient.

Providing each individual patient with customized information was seen as an important factor. Patients’ input in conversations with healthcare providers was vital when determining personalized treatment and care. This was expressed as follows by a nurse:

*But if a measure is in line with what the patient wants*,* that’s often the best thing*,* rather than*,* eh*,* us checking the level less often or a bit more often than the patient wants in a way*,* so the measure is often best if it is in line with what the patient thinks is important there and then*,* and that they feel okay. And some of them hardly notice fluctuations in their blood glucose level*,* while others feel very poorly because of such fluctuations* (Participant 2).

Some participants emphasized the importance of maintaining strict glycemic control for certain patients, as they had observed the challenges faced by patients who struggled to relinquish tight glycemic control. Some said that they would rather adjust the patients’ blood glucose with insulin than restrict what they could eat. It could sometimes be a challenge for the healthcare professionals to see patients refusing to eat sugary foods because of their lifetime experience of following a strict glycemic control regime. One of the dietitians said:

*I think that we are really good at accommodating the patients*,* on what they themselves want. If they’re a bit rigid and inflexible about those things*,* then we let them be like that as well … you kind of keep in step with them*,* help them to feel okay … be able to eat what they want as far as possible. That’s where I think that we do our best* (Participant 1).

They also experienced that some symptoms of diabetes can mimic symptoms the patients can suffer from. One nurse put it as follows:

*It’s kind of*,* I don’t know … the diabetes and the palliation are like*,* there are so many things that merge together in one way or another (Participant 9).*

### An individualized approach

The healthcare professionals emphasized tailoring and individualizing diabetes treatment according to each individual’s unique circumstances based on their life expectancy, difficult blood glucose control, and multidisciplinary collaboration.

#### Tailoring diabetes care to life expectancy

Some of the patients were hospitalized for a short period, while others were hospitalized for symptom relief and were discharged to their homes. If the patients were discharged to their own homes with a life expectancy of several months or even years, they usually followed the previously prescribed treatment of blood glucose-lowering medication and blood glucose measurements as they had before they were admitted. One of the nurses described this as follows:

*If medicines have been discontinued and they are eating very little*,* then we don’t check the blood glucose level if they are only expected to have a very short time left*,* hours or days*,* but if it’s someone who we expect to live for weeks or months*,* then we do check it. So there can be that difference* (Participant 2).

The participants pointed out that medication and measuring blood glucose were discontinued when the patients stopped eating and drinking. However, they did not have specific procedures to follow. One physician said the following:

*I think like*,* I do at least*,* I think that when the patient is no longer eating and drinking*,* then I believe it’s meaningless to give them insulin. It will*,* you know … if they get diabetic ketoacidosis*,* then so what? It makes no difference*,* in my view* (Participant 11).

Another physician said that insulin treatment was maintained for somewhat longer if the patient had type 1 diabetes:

*That we continue the insulin treatment a little longer in such case and are perhaps a bit more concerned about glycemic control*,* then maybe … but it’s only a gut feeling I have*,* that we are a bit stricter about that or that we maybe keep up this strict type 1 diabetes treatment a bit longer then* (Participant 12).

Some noted that they tended to pay more attention to unpleasant symptoms and side effects caused by medications administered to treat other comorbidities, such as high blood pressure or high cholesterol, than the patient’s diabetes. They were keen to take a critical look at the list of medicines to eliminate discomfort and try to improve the quality of life.

#### More difficult blood glucose control

Treatment with corticosteroid was mentioned as a challenge because it tends to make glycemic control more unstable, more fluctuating, and difficult to manage. The consequence of this was more measuring of blood glucose levels or insulin injections. Corticosteroids increase blood glucose levels and measuring blood glucose is therefore essential. One nurse said:

*It’s not unusual for someone to be given steroid treatment that often has a very strong effect on the blood glucose level and that can be difficult to control* (Participant 3).

During the analysis it became clear that it could be difficult to manage blood glucose when treating patients with diabetes in palliative care, and that each patient could react differently to the same blood glucose levels. The healthcare professionals stressed that the number of measurements was individualized based on the patient’s requirements, how stable their blood glucose was, and life expectancy. One of the dietitians put it as follows:

*How high a blood glucose level people tolerate is variable*,* you know – some of them are very affected by it*,* while others can have a pretty high level without seeming to be so very troubled by it* (Participant 8).

#### A multidisciplinary approach enables individualization

Multidisciplinary teams play a crucial role in palliative care, which promotes an individualized approach. The participants stressed that the diverse expertise of the healthcare professionals involved contributed to tailoring care based on their unique perspectives. By collaborating with one another, they provided holistic support. They emphasized that they had regular weekly meetings at which the voices of all team members were given priority and emphasis was placed on the healthcare professionals’ own experience. Almost all the participants stated that the way they worked in palliative care was unique in terms of cooperation between the various professional groups. One of the nurses put it as follows:

*Everyone is present at least several times a week … sometimes we also have multidisciplinary meetings if there are some patients we find it especially challenging to help* (Participant 2).

### Diabetes in the background

The healthcare professionals had a modest focus on diabetes treatment. Consideration of diabetes was not prominent, but rather another aspect of the health of the patient they had to be aware of. The lack of attentiveness to technical tools and treatment choices, diabetes guidelines, and care-decisive competence underscored that awareness of the diabetes treatment was not prominent in palliative care.

#### Symptom control is the main issue

The participants emphasized that the treatment of diabetes in palliative care focused on trying to avoid blood glucose spikes. Symptom control was the main issue, while the diabetes guidelines were more in the background. The healthcare professionals addressed patients’ most troublesome symptoms, such as pain, dry mouth, shortness of breath, and anxiety. Diabetes received limited attention and was often considered a secondary rather than a primary concern in palliative care. One physician said:

*I don’t feel that it’s been like … I sort of feel that diabetes has always been a kind of*,* yes*,* a secondary diagnosis. I feel that it’s never been*,* like*,* the primary focus* (Participant 12).

Thus, the healthcare professionals focused more on the symptom pressure of the patient than on their diabetes and treatment options, described as follows by one nurse:

*It’s not the diagnosis that matters*,* it’s more the symptom burden* (Participant 3).

Still, the healthcare professionals expressed different opinions to whether patients should continue with dietary restrictions because of their diabetes. Some participants were concerned that the patients should have a diabetic diet to adapt the food to their diabetes. One nurse expressed it like this:

*When it comes to diet*,* for instance*,* it is very different how we nurses practice this matter as well*,* so some have probably tried to be on the stricter side because they want to do everything possible in terms of food*,* while others think that the national guidelines should be taken notice of (Participant 4*).

Another nurse emphasized that they encouraged all patients to eat what they wanted to:

*“I think that in relation to diet*,* we can have the kitchen staff to accommodate wishes to get adapted food to each individual (Participant 3).*

Given the fact that many patients with advanced disease experienced reduced appetite, they emphasized ensuring that the food provided did not exacerbate any existing issues for the patients and instead tried to enhance the patients’ well-being. However, some of the healthcare professionals highlighted that they explained to patients that eating unhealthy food that caused too much discomfort might not be worthwhile. As an alternative, they highlighted getting enough energy to function during the day or eating to experience joy without feeling guilty about it. One dietitian put it as follows:

*That they get enough energy from the food without this creating problems for them*,* but that they nonetheless get enough energy to manage to do what they want to do* (Participant 8).

#### Lack of training in technical tools for diabetes treatment

Some healthcare professionals stated that they had only received guidance in the use of glucose sensors from the patients. The patients were mostly able to use the glucose sensors themselves. None of the participants said that they had been trained in the availability and use of new technical tools by other healthcare professionals. One nurse found sensors difficult to manage:

*I must say that I find it a bit difficult with those sensor things when I haven’t been given training*,* and maybe I sound like an 80-year-old*,* but I think it’s difficult*,* you know (Participant 4).*

Another challenge was that, if the sensor was connected to a personal mobile phone rather than a reader, the healthcare professionals needed to have access. The patients then had to share their private login codes with them. Likewise, without previous experience of using insulin pumps it could be easier to treat patients with insulin pens versus insulin pumps because of the lack of experience.

*With poorly patients*,* when they deteriorate …*,* then I think that it’s almost easier with a pen … of course*,* that also has to do with my experience with insulin pumps* (Participant 3).

#### Lack of significant diabetes guidelines and care-decisive competence

Some of the participants said that they did not have much prior knowledge of diabetes. They pointed out that they relied on previous experience of treating diabetes when they worked with diabetes patients in palliative care. It was desirable to have some knowledge of diabetes treatment beforehand. One nurse said:

*So*,* there’s a lot of concerns with diabetes patients here that you have to be aware of*,* you should preferably have some prior knowledge before starting here (Participant 9).*

The care that the healthcare professionals provided to patients with diabetes in palliative care depended on their individual experience, knowledge, and competence as regards diabetes treatment.

The study revealed that healthcare professionals lacked guidelines and procedures for diabetes treatment in palliative care, and that they primarily relied on clinical judgment and prior knowledge when treating these patients. One physician put it as follows:

*It’s not like a written procedure*,* more that we are so few doctors here and that we have such extensive experience that we do it*,* but slip-ups can occur because we don’t have written procedures for it. We don’t have procedures for it*,* it’s not written down in one of those*,* in a plan*,* you know*,* so you just have to remember it* (Participant 12).

Some of the participants experienced that it might actually be difficult to have guidelines that suited everyone because of the different needs the patients have. One nurse said the following:

*It’s a bit difficult to have guidelines that are suitable for everyone and there are a lot of needs that must be considered*,* such as*,* well*,* how much do they eat and how stable was their blood glucose level before*,* what do they usually do (Participant 2).*

## Discussion

This study explored healthcare professionals’ experiences when providing palliative care for patients with diabetes. The findings revealed three main themes: the first emphasized quality of life as the main focus, the second accentuated individualized care, and the third demonstrated that diabetes was recognized as being somewhat in the background.

The healthcare professionals’ main focus was on making sure that the patients had the best possible quality of life. They experienced that relational care and sufficient time for conversation could contribute to increasing the patient`s wellbeing. They were attentive to adapting to the patients and families’ wishes to promote the best treatment. The Norwegian general guidelines for palliative care recommend utilizing advanced care planning to give patients and their families an opportunity to express treatment preferences and receive appropriate symptom management [16]. Although diabetes-specific guidelines are lacking in palliative care in Norway, international literature describes challenges and strategies for end-of-life care of people with diabetes [7]. Healthcare professionals can help patients with diabetes by developing suitable care plans based on their values and preferences to enhance their quality of life and help them avoid bothersome terminal treatment. To avoid troublesome terminal treatment, healthcare professionals should discuss the patient’s wishes and preferences in relation to the treatment of their diabetes early in the palliative phase.

In the present study, the healthcare professionals stressed that patients were urged to eat whatever they wanted. Food plays an important part in social interaction and can give a feeling of pleasure and enjoyment [17]. The patients were therefore encouraged to eat both for pleasure and to get enough energy to be able to do the things that were important to them. However, some of the healthcare professionals took a slightly different approach as regards diets for patients with diabetes. They stated that they tended to be on the stricter side. This attitude differed from the standpoint of the dietitians, who communicated that they considered this an old-fashioned attitude. They claimed that healthcare professionals at the unit could adjust the patients’ blood glucose levels using insulin rather than restricting what they could eat. Although dietitians ought to have a more pivotal role in the treatment and care of palliative care patients with diabetes, access to a dietitian’s expertise is unfortunately depending on the level of health care one is receiving and the organization of the palliative care service. In the literature, it is stated that a flexible approach to food choices is beneficial for patients with diabetes in palliative care [10, 18, 19]. It is also pointed out, however, that it may be difficult for some patients not to meet their diabetes treatment goals at this point in life, as this may be perceived as giving up [20]. The participants stated that they had experienced that patients had found it challenging to relinquish strict glycemic control. These patients often needed more reassurance and dialog.

Previous research shows that patients may benefit from healthcare professionals having some diabetes expertise so that they can better treat diabetes ailments and support the patients in a palliative setting [10, 17, 21]. In high-quality palliative care providing the healthcare professionals working in these settings with basic knowledge on handling diabetes in a palliative phase is important for the patients` feeling of optimal care and safety. Healthcare professionals should recognize that the burden of symptom severity is crucial for the patients in a palliative phase and thus provide optimal diabetes care to avoid unnecessary symptom severity [22]. Our findings highlight that the healthcare professionals wanted to provide patient care in a holistic perspective, and that communication and care planning that is tailored to patients’ preferences plays a crucial role. Patients want healthcare professionals to listen and acknowledge their own expertise in managing their diabetes [21]. Nevertheless, the results in the present study underscore that, when the patients’ health deteriorated and their life expectancy decreased, healthcare professionals became more relaxed about glycemic control. This is somewhat in contrast to previous research on the experiences and care preferences of people with diabetes at the end of life. It underscores that patients prefer to be monitored closely to avoid hypoglycemia and hyperglycemia and to promote their comfort [23]. However, our results show that the healthcare professionals were more likely to follow up the patient’s more carefully with frequent blood glucose measurements when the patients started on corticosteroids. This is in line with other literature warning that this medication can make blood glucose levels more unstable and harder to treat [24]. To reduce the risk of high blood glucose levels during the day while using corticosteroids, patients could benefit from more careful blood glucose measurement and being given diabetes medication such as insulin in the morning [20].

Symptom assessment and management is an essential component of palliative care, and the findings from our study accentuate that the healthcare professionals placed significant emphasis on treating symptoms in a holistic way. Multidisciplinary teams play a crucial role in palliative care and promote individualized approaches. The participants in our study found this way of working to be satisfactory and necessary to provide the best possible care for the patients. Such diverse expertise among healthcare professionals can contribute to tailoring care based on their unique perspectives [16]. However, it was also evident that some of the participants displayed a lack of professional awareness as regards diabetes. They neither recognized nor found the connection between diabetes and other comorbidities to be important. They were more concerned with symptom control and alleviating troublesome symptoms such as pain and nausea. However, it was emphasized that the goals for diabetes management in end-of-life care need to focus on comforting and controlling distressing symptoms through simplified treatment regimens that promote quality of life [9]. Managing risks and benefits, while at the same time preventing hypo- or hyperglycemia situations is a continuing process [21].

The healthcare professionals nonetheless had a modest focus on diabetes and tended to regard it as a secondary diagnosis. Their attentiveness to diabetes treatment and the effect diabetes had on other symptoms was not prominent. This has also been shown in other research that has indicated that healthcare professionals may not devote enough attention to the patients’ diabetes when they are admitted for other diseases [23]. Moreover, healthcare professionals may not take into account diabetes-related factors that concur with other symptoms if they fail to measure blood glucose consistently [23]. In this respect, healthcare professionals’ prior experience and expertise may affect the diabetes treatment given. The Diabetes UK End of Life Guidance for Diabetes Care emphasizes that it might benefit the patients if healthcare professionals are more attentive, reflect on their own knowledge and professional development needs [17, 18]. Apart from having enough knowledge to capture the severity of symptoms themselves, they might consider asking for support by endocrinologists or specialist nurses when needed.

The findings from our study indicate that healthcare professionals lacked training in technical tools for diabetes treatment. Such tools, e.g., continuous glucose monitoring (CGM), have the potential to offer a less bothersome approach to monitoring blood glucose [25]. However, some of the participants found using it rather difficult because of a lack of training. Moreover, they stated that needing to know the patients’ mobile phone access codes when patients used glucose sensors was invasive in relation to the patients’ privacy. Guidelines from the United Kingdom (UK) state that, when patients cannot control the CGM themselves, it is not necessary to discontinue its use if the healthcare professionals are given adequate training in how to use it [17]. Findings from a previous case study show that a pre-calibrated CGM combined with a closed-loop system could be a safer and less bothersome way to measure blood glucose, and that it might help to individualize and follow the patient’s glucose goals [25].

To ensure good diabetes treatment in palliative care, it would be beneficial if healthcare professionals were well trained in managing diabetes [17, 20]. We found that diabetes treatments, such as measurement and insulin injections, were discontinued when the patients were no longer eating and drinking, regardless of the patient’s diabetes type. This is in contrast to guidelines from the UK, which suggest that insulin should never be discontinued for those who have type 1 diabetes [17]. Giving insulin a bit longer if the patient has type 1 diabetes might reduce the risk of unpleasant symptoms. Our results also show that some of the healthcare professionals did not see it as a problem that patients developed ketoacidosis as they approached death. This is somewhat unexpected as previous research has shown that patients may find hypo- and hyperglycemia quite uncomfortable [17, 23].

The importance of providing condition-specific, end-of-life care is described in the literature [26]. The absence of diabetes-specific guidelines in palliative care, as shown in our study, may result in varying treatment approaches, depending on healthcare professionals’ prior experiences of diabetes in palliative care. In order to facilitate excellent quality care, it is important to provide accessible and concise clinical recommendations for healthcare professionals [17].

### Study strengths and limitations

This study made it possible to explore and get more insights in the healthcare professionals` experiences of providing palliative care to patients with both type 1 and type 2 diabetes. Although these findings cannot be transferred directly to other palliative contexts, they might shed some light on challenges also experienced in other palliative care units. Their experiences may guide the healthcare professionals to give a more holistic palliative care in the future. The credibility of the study is strengthened by the research team comprising nurses with extensive experience of diabetes as well as palliative care. Moreover, it is a strength that two of the researchers have considerable experience of qualitative data analysis. Preconceived notions about diabetes, palliative care, and qualitative methods proved positive when developing the interview guide. Such expertise ensures that relevant data were collected and that appropriate follow-up questions were found that it would be natural to ask in the interview setting. Although one of the interviews lasted only 17 min this informant managed to enrich the data with more variation and insight on the topic. It was a strength that a pilot interview was conducted since this made it possible to further adjust the questions. On the other hand, despite considerable clinical experience, a preunderstanding of treating diabetes and palliative care patients may also be a limitation in the analysis process. To ensure that preconceptions did not take up too much space, reflexive discussions were held throughout the analysis process. The extensive clinical experience of the research group in the field of diabetes care and palliative care facilitated reflection during the analysis, as well as awareness of our assumptions in this process. However, it might be a limitation that we did not include other professions, such as physiotherapists and endocrinologists.

## Conclusion

The treatment approach for patients with diabetes in palliative care primarily emphasizes symptom relief and personalized adjustments, sometimes leading to the diabetes diagnosis being pushed into the background. End-of-life treatment of diabetes depends on the healthcare professionals’ experience, professional views, and the circumstances of each individual. The lack of diabetes-specific guidelines in palliative care contributes to different approaches. Further research is warranted to help improve clinical practice.

## Electronic supplementary material

Below is the link to the electronic supplementary material.

Supplementary Material 1

## Data Availability

Due to personal data protection legislation and legal restrictions related to confidentiality, the data is not publicly available as the study participants have not explicitly been informed about, nor approved data sharing when the data were gathered (see approval for processing of personal data from the Norwegian Agency for Shared Services in Education and Research (SIKT) (ref. 550782). However, the committee allows us to include collaborators if the research questions align with the research question and purpose of the study that the participants have been informed about. If someone wants to request the data from this study, please contact the corresponding author.

## Abbreviations

CGM: Continuous Glucose Monitoring

## References

[1] WHO. The World Health Organization Quality of Life (WHOQOL) [Internet]. World Health Organization. 2012 [cited 15. mars 2023]. https://www.who.int/publications/i/item/WHO-HIS-HSI-Rev.2012.03

[2] WHO. Palliative care, [Internet] World Health Organization., 2020 [cited 20. June 2023]. https://www.who.int/news-room/fact-sheets/detail/palliative-care

[3] Akershus universitetssykehus. *Lindrende behandling* [Palliative Care] [Internett] [cited 18. December 2023] Available from: Lindrende behandling - Akershus universitetssykehus HF (ahus.no).

[4] Ko C, Chaudhry S. The need for a multidisciplinary approach to cancer care. J Surg Res. 2002;105(1):53–7. 12069502 10.1006/jsre.2002.6449

[5] International Diabetes Federation. IDF Diabetes Atlas. 2021. https://diabetesatlas.org/atlas/tenth-edition/

[6] Bettencourt-Silva R, Aguiar B, Sá-Araújo V, Barreira R, Guedes V, Ribeiro M, Carvalho D, Paulo MS. Diabetes-related symptoms, acute complications and management of diabetes mellitus of patients who are receiving palliative care: a protocol for a systematic review. BMJ open. 2019;9(6):e028604. 10.1136/bmjopen-2018-028604. 31203247 10.1136/bmjopen-2018-028604 PMC6589017

[7] Dunning T, Martin P. Palliative and end of Life Care of people with diabetes: issues, challenges and strategies. Diabetes Res Clin Pract. 2018;143:454–63. 10.1016/j.diabres.2017.09.018. 29097287 10.1016/j.diabres.2017.09.018

[8] Hindson D, Colliety P, Williams P, Shawe J. An audit review of end-of-life care for inpatients with diabetes. J Diabetes Nurs. 2012;16(4):154–59.

[9] Munshi MN, Florez H, Huang ES, Kalyani RR, Mupanomunda M, Pandya, et al. Management of diabetes in long-term care and skilled nursing facilities: a position Statement of the American Diabetes Association. Diabetes Care. 2016;39(2):308–18. 10.2337/dc15-2512. 26798150 10.2337/dc15-2512 PMC5317234

[10] Dunning TL. Palliative and End-of-Life Care: vital aspects of holistic diabetes care of older people with diabetes. Diabetes Spectr. 2020;33(3):246–54. 10.2337/ds20-0014. 32848346 10.2337/ds20-0014 PMC7428665

[11] Sharma A, Sikora L, Bush SH. Management of diabetes Mellitus in adults at the end of life: a review of recent literature and guidelines. J Palliat Med. 2019;22(9):1133–38. 10.1089/jpm.2018.0614. 30892135 10.1089/jpm.2018.0614

[12] Tong A, Sainsbury P, Craig J. Consolidated criteria for reporting qualitative research (COREQ): a 32-item checklist for interviews and focus groups. Int J Qual Health Care. 2007;19(6):349–57. 10.1093/intqhc/mzm042. 17872937 10.1093/intqhc/mzm042

[13] Braun V, Clarke V. Using thematic analysis in psychology. Qualitative Res Psychol. 2006;3(2):77–101. 10.1191/1478088706qp063oa.

[14] Braun V, Clarke V. *Thematic analysis: a practical guide to understanding and doing*, 2021;1.Ed. Thousand Oaks: SAGE.

[15] Braun V, Clarke V. Conceptual and design thinking for thematic analysis. Qualitative Psychol. 2022;9(1):3–26. 10.1037/qup0000196.

[16] Helsedirektoratet. (2015). *Nasjonalt handlingsprogram med retningslinjer for palliasjon i kreftomsorgen [nettdokument].* Oslo: Helsedirektoratet (updated 14. October 2019((cited 01. June 2023). https://www.helsedirektoratet.no/retningslinjer/palliasjon-i-kreftomsorgen-handlingsprogram

[17] Diabetes UK. End of Life Guidance for Diabetes Care, 4. ed. [Internet] Diabetes UK, 2021. https://diabetes-resources-production.s3.eu-west-1.amazonaws.com/resources-s3/public/2021-11/EoL_TREND_FINAL2_0.pdf#:~:text=Generic%20guidance%20on%20end%20of%20life%20care%20also,and%20education%20of%20the%20healthcare%20workforce%20is%20included1

[18] Smyth T, Smyth D. Considering palliative and end-of-life care for people with diabetes. Pract Nurs. 2011;22(11). 10.12968/pnur.2011.22.11.578.

[19] Claydon A, Spencer K. Improving diabetes care at the end of life. Nurs Standard. 2015;30(6):37–42. https://doi.org/. 10.7748/ns.30.6.37.s45 26443175

[20] James J, Dying D, Diabetes. Practical Diabetes. 2021;38(3):37. 10.1002/pdi.2340.

[21] Dunning T, Savage S, Duggan N, Martin P. Palliative and end of life care for people with diabetes: a topical issue. Diabetes Manage. 2014;4(5):449–60. (openaccessjournals.com). end-of-life-care-for-people-with-diabetes-a-topical-issue.pdf.

[22] Quill TE, Abernethy AP. Generalist plus specialist palliative care–creating a more sustainable model. N Engl J Med. 2013;368(13):1173–5. 10.1056/NEJMp1215620. 23465068 10.1056/NEJMp1215620

[23] Savage S, Duggan NM, Dunning T, Martin P. The experiences and Care preferences of people with diabetes at the end of life: a qualitative study. J Hospice Palliat Nurs. 2012;14:293–302. 10.1097/NJH.0b013e31824bdb39.

[24] Alqabandi N, Haywood A, Kindl K, Khan S, Good P, Hardy J. Managing diabetes at the end of life – a Retrospective Chart audit of two Health providers in Queensland, Australia. Progress Palliat Care. 2019;27(2):51–7. 10.1080/09699260.2019.1611721.

[25] Boughton CK, Bally L, Hartnell S, Wilinska M, Coll AP, Evans M, Stettler C, Hovorka R. Closed-loop insulin delivery in end-of-life care: a case report. Diabet Med. 2019;36(12):1711–14. 10.1111/dme.13974. 31002426 10.1111/dme.13974 PMC6900195

[26] James J. Death, dying and diabetes: The importance of providing condition-specific end-of-life care. [Internet] J Diabetes Nurs. 2014;18(8):308–16. https://diabetesonthenet.com/journal-diabetes-nursing/death-dying-and-diabetes-the-importance-of-providing-condition-specific-end-of-life-care/

