# Supplementary material for: Healthcare professionals’ experiences of providing palliative care for patients with diabetes – a qualitative study
Source: BMC Palliat Care. 2024 Oct 10;23:241. doi: 10.1186/s12904-024-01567-4 (PMC11468092; doi:10.1186/s12904-024-01567-4)
Supplement: Supplementary file 1 — Supplementary Material 1 [file 12904_2024_1567_MOESM1_ESM.pdf]

Interview guide	
**Topics and main questions**	**In-depth follow-up questions (probe questions) to extend the conversation**
Experiences	
Can you describe your experiences with palliative care?	- Which patient groups do you have the most experience with?<br>- How long have you been working with palliative care patients?<br>- Have you only worked in this department or have you also worked elsewhere?
Can you describe your experience of treating people with diabetes?	
Can you say a little more about your experience with palliative care for people with diabetes?	- Can you say a little more specifically about which patients these have been? What was the main diagnosis?<br>- What was their situation like?<br>For instance: What type of diabetes did they have? Had they had diabetes for a long/short period of time? Were they younger or older?
Are there specific guidelines in your department/hospital for palliative care related to people with diabetes?	- Where do you find these guidelines?<br>- Are they easily accessible?<br>- Does everyone in your workplace know about them?
Can you describe in more detail what is the current practice in the palliative care field?<br><br>Can you describe what it's like here where you work?	- Do you know if current practice is different elsewhere?<br>- Is there great variation in the treatment of different patients?<br>- Is everyone treated equally in your workplace?

How do you work in multidisciplinary teams?	- How do you safeguard multidisciplinary work?<br>- Which professional groups are involved?<br>- Can you describe in a little more detail how you work?<br>- Can you give examples?
Do you have any experience with palliative patients who have diabetes as an additional diagnosis making it challenging as a healthcare professional?	- Are you comfortable working with this patient group?
**Symptom relief**	
Can you say a little about which interventions you think provide the best symptom relief in people with diabetes in palliative care?	- How do you distinguish between the different symptoms (whether they are related to the diagnosis that requires palliative care or hypoglycemia or hyperglycemia)?
Can you tell a little bit about how you assess patients?	- Can you talk about which forms/tools you use?<br>- Do you use any questionnaires or instruments (if so, which ones)?<br>- How do you feel that the assessments work?<br>- Do you follow up with reassessment and/or evaluation of implemented measures?
Can you say a little bit about how you assess the patient's quality of life?	- Do you use any tools of your own?
Glycemic control	
Can you say a little bit about how current practice facilitates satisfactory glycemic control in palliative care?	- Are there measures that stand out as particularly good?<br>- How would you describe what is "satisfactory?"<br>- Can you give some examples?
Can you share your experiences with blood glucose monitoring in palliative care?	- How do you individualize the number of times you measure blood glucose?

	- Do you find that there is a difference in the number of measurements based on the type of diabetes the patients have?
Can you tell a little bit about what considerations you take with regards to other treatments for blood glucose?	- Can you say a little about other treatments? Describe.<br>- How do you modify nutrient intake with the need for insulin?<br>- Can you describe how you adapt your measurements of blood glucose and insulin needs to your glucose levels?
Describe the assessments that healthcare professionals make to strive for balanced blood glucose?	- Is it done in the same way by everyone in the department?
Can you say a little about the difference in the treatment of people with diabetes in palliative care in relation to their additional diagnoses?	- What do you think about any other medication that can increase blood glucose?<br>- Can you give some examples of medication that can increase blood glucose?
Information	
In general, can you say a little bit about the information provided to patients and their families in palliative care concerning the diabetes diagnosis?	- Do you find that the patient and their next of kin get the information they need?<br>- What information do you provide for the patients/relatives?<br>- Does the information provided vary based on how far along the patient is in their course of palliative care?
How do you perceive the patients' need for conversation and information about their diabetes?	- Will their needs be met?<br>- Will enough time be set aside for conversation?<br>- Are there any other reasons why patients do not receive or have their needs met for counseling?<br>- How are relatives involved?

Quality of life	
Can you talk a little bit about how you perceive the quality of life of patients with diabetes in palliative care?	- Say a little about how you facilitate the best possible quality of life.<br>- What do you feel is the best intervention to increase the quality of life of people with diabetes in palliative care?
Do you find that there are any measures that improve the quality of life of these patients?	
**Technical tools in the treatment of diabetes**	
What experience do you have with the use of CGM (Continuous Glucose Monitoring) in palliative patients?	- When comparing CGMs against finger glucose readings: Do you see any advantages/disadvantages of the different methods?<br>- How do you find that this tool works for glycemic control?
What experiences do you have with insulin pumps?	- What do you think about patients having a pump in palliative care?<br>- Compared to pen therapy, how do you experience pump therapy in palliative care?<br>- Do you receive guidance in the use of an insulin pump for patients in palliative care?
For physicians	
Medications	
Can you say a little about pharmacological interventions that are different when people with diabetes are in palliative care compared to those who do not have diabetes?	- Are there other medications that affect treatment choices?<br>- Say a little about your experiences regarding the frequency of blood glucose monitoring in palliative care
Can you say a little about your experiences with insulin therapy in people with type 1 diabetes in palliative care?	- Does insulin therapy vary depending on how far along the patients are in the course of palliative care?<br>- Are there big differences?
Can you say a little about your experiences with insulin therapy in people with type 2 diabetes in palliative care?	- Does insulin therapy vary depending on how far along

	the patients are in the course of palliative care?<br>- Are there big differences?
Can you say a little about how you include other professional groups in your choice of treatment?	
When do you consider discontinuing insulin therapy for patients at the end of their life?	- What criteria form the basis for discontinuing?
For nurses	
Can you tell a little about what measures you have experienced that provide the best possible symptom relief for patients with diabetes in palliative care?	- Which non-pharmacological interventions give the best effect on glycemic control?<br>- How do you distinguish between symptoms such as hypoglycemia and hyperglycemia from other symptoms palliative care patients may have?
Can you say a little about how nurses can contribute to increasing the quality of life in these patients?	
Can you say a bit about the difference between the treatment of people with type 1 diabetes and type 2 in palliative care?	- Is there a big difference in treatment and follow-up?<br>- Can you say a little about your experiences with insulin therapy for people with type 1 diabetes?<br>- Can you say a little about your experiences from the treatment of type 2 diabetes?<br>- Can you say a little about non-pharmacological interventions for patients with type 1 and type 2 diabetes?
Can you say a little bit about whether you consider that other professional groups include you in treatment choices?	- Are you included?
**For dietitians**	
Can you say a little about what dietary advice you would give to a patient with diabetes in palliative care?	- Do you have any experience on which measures work best to prevent hypo- and hyperglycemia?<br>- How do you adapt/adjust dietary advice?<br>- Do you find that there is a big difference between advice given to palliative patients

	without diabetes than to patients with diabetes?<br>- How do you feel that palliative care affects the advice you give to people with diabetes?
Can you say a little about nausea and appetite in palliative care patients?<br>How do you feel about nausea and appetite in these patients?	- Have you experienced that there are some measures that work better than others regarding appetite and nausea for patients?
Can you say a little about the advice you give to people with diabetes in palliative care in relation to the choice of nutritional supplements/replacements?	- Do you give different advice to people with diabetes than those without?<br>- Say a little about the initiatives you think provide the best symptom relief in these patients.
